# Wnt-Independent SARS-CoV-2 Infection in Pulmonary Epithelial Cells

**DOI:** 10.1128/spectrum.04827-22

**Published:** 2023-06-27

**Authors:** Alexey Koval, Jiabin Xu, Nathalia Williams, Mirco Schmolke, Karl-Heinz Krause, Vladimir L. Katanaev

**Affiliations:** a Department of Cell Physiology and Metabolism, Translational Research Centre in Oncohaematology, Faculty of Medicine, University of Geneva, Geneva, Switzerland; b Department of Microbiology and Molecular Medicine, Faculty of Medicine, University of Geneva, Geneva, Switzerland; c Department of Pathology and Immunology, Faculty of Medicine, University of Geneva, Geneva, Switzerland; d Institute of Life Sciences and Biomedicine, Far Eastern Federal University, Vladivostok, Russia; University of Sussex

**Keywords:** SARS-CoV-2, Wnt, lung epithelia, clofazimine, Wnt pathway inhibitors, Wnt signaling, drug discovery

## Abstract

The Wnt signaling pathway within host cells regulates infections by several pathogenic bacteria and viruses. Recent studies suggested that severe acute respiratory syndrome coronavirus 2 (SARS-CoV-2) infection depends on β-catenin and can be inhibited by the antileprotic drug clofazimine. Since clofazimine has been identified by us as a specific inhibitor of Wnt/β-catenin signaling, these works could indicate a potential role of the Wnt pathway in SARS-CoV-2 infection. Here, we show that the Wnt pathway is active in pulmonary epithelial cells. However, we find that in multiple assays, SARS-CoV-2 infection is insensitive to Wnt inhibitors, including clofazimine, acting at different levels within the pathway. Our findings assert that endogenous Wnt signaling in the lung is unlikely required or involved in the SARS-CoV-2 infection and that pharmacological inhibition of this pathway with clofazimine or other compounds is not a universal way to develop treatments against the SARS-CoV-2 infection.

**IMPORTANCE** The development of inhibitors of the SARS-CoV-2 infection remains a need of utmost importance. The Wnt signaling pathway in host cells is often implicated in infections by bacteria and viruses. In this work, we show that, despite previous indications, pharmacological modulation of the Wnt pathway does not represent a promising strategy to control SARS-CoV-2 infection in lung epithelia.

## INTRODUCTION

The Wnt signaling pathway controls numerous steps in human embryonic development and adult physiology (1, 2). Improper Wnt pathway activation underlies oncogenic transformation and progression (3, 4). As in many other organs, Wnt signaling controls both development and homeostasis in the lungs (5, 6), and different aberrations in the normal Wnt pathway levels underlie a number of lung pathologies, such as asthma, chronic obstructive pulmonary disease (COPD), or cancer (5, 7, 8). These features make the Wnt pathway a highly desirable target for a number of drug discovery programs. Multiple approaches are undertaken to develop novel Wnt pathway inhibitors, such as dedicated high-throughput screening (HTS) assays of chemical libraries (9, 10) or natural products of terrestrial and marine origin (11, 12), as well as repurposing of approved drugs (13, 14). Among the latter, the antileprotic and antituberculosis drug clofazimine has been identified by us as a specific inhibitor of Wnt signaling. Clofazimine inhibits cancer cell proliferation and *in vivo* tumor growth in a range of cancer types, such as triple-negative breast cancer (TNBC), hepatocellular carcinoma (HCC), colon cancer, and ovarian cancer (15–17).

COVID-19, caused by a coronavirus infection, is an acute respiratory disease with a broad clinical spectrum that ranges from mild to severe manifestations and has caused 6.8 million deaths up to March 2023 (https://www.who.int/publications/m/item/weekly-epidemiological-update-on-covid-19---30-march-2023). As there are currently no widely available specific antiviral therapies for coronaviruses in humans (18), the urgent and utmost need for the search for efficient treatment regimens for COVID-19 is obvious. As for oncology indications, repurposing of approved drugs is a popular approach for the rapid development of anti-infection therapies. When applied to severe acute respiratory syndrome coronavirus 2 (SARS-CoV-2), this approach led to the identification of several promising compounds (19), among them, recently, clofazimine (20). Clofazimine has been shown to be effective in a number of SARS-CoV-2 assays *in vitro* and in animal models and has revealed the capacity to block several steps of the coronavirus infection, such as the initial viral entry, spike-mediated cell fusion, viral RNA synthesis, and the unwinding activity of the SARS-CoV-2 helicase (20). This multiplicity of targets may indicate that clofazimine acts not at a given viral component, but at a host cell component repeatedly used by the coronavirus. As the effective concentrations of clofazimine in these different viral assays (50% inhibitory concentrations [IC_50_s] ranging from 1 to 10 μM) are identical to the effects of the drug we have previously observed on Wnt signaling and Wnt-dependent cellular activities (15–17), the starting hypothesis of the current work was that the inhibitory effect of clofazimine on the coronavirus infection is mediated by suppression of the endogenous Wnt signaling by the drug. This initial hypothesis is further enforced by a recent report that β-catenin, a key component of the Wnt pathway, is important for SARS-CoV-2 infection (21). If confirmed, the important role of the endogenous host cell Wnt pathway in SARS-CoV-2 infection would increase the list of infections dependent on this signaling. Indeed, Wnt signaling previously emerged in a number of studies as an important player in different pathogen infections and the resulting human diseases. Regarding pathogenic bacteria, cases of infection and disease caused by, e.g., Clostridium difficile in the colon (22), Helicobacter pylori in the stomach (23), or Mycobacterium tuberculosis in the lungs (24) have all been shown to depend on Wnt signaling in these organs. Several viruses, such as the hepatitis virus B (25) or C (26), herpesviruses (27), or HIV (28), are also known for their dependence on and remodeling of the Wnt signaling, which contributes to the viral progression and/or pathological consequences of the infection (29).

In this work, we assessed Wnt signaling and its role in SARS-CoV-2 infection in lung epithelia. Surprisingly, we find that the SARS-CoV-2 infection in lung cells is independent from Wnt signaling and that pharmacological modulation of the Wnt pathway does not provide a promising route to control the SARS-CoV-2 infection.

## RESULTS AND DISCUSSION

We set out to investigate the effects of clofazimine and other anti-Wnt compounds on physiological Wnt signaling in lung cancer-derived epithelial cells and their relationship with the effectiveness of SARS-CoV-2 infection. We began with an analysis of the Wnt signaling effects on the following human lung epithelium-derived cell lines: A549 (adenocarcinoma alveolar basal epithelial cells) and Calu-3 (non-small-cell lung cancer cell line). Calu-3 is a well-established “workhorse” of SARS-CoV-2 research since it is one of the few lines naturally expressing ACE2 (30). A549 cells do not provide native ACE2; therefore, we used a stable line, A549-ACE2-TMPRSS2, expressing both ACE2 and TMPRSS2, the factors essential for viral entry (31). Using a lentivirus-delivered TopFlash reporter construct, we produced a Calu-3-derived line stably infected with this Wnt reporter; the A549-ACE2-TMPRSS2 cells were transiently transfected with TopFlash for monitoring of the Wnt signaling levels. For simplicity, we refer to the A549-ACE2-TMPRSS2 and Calu-3-TopFlash cell lines as simply A549 and Calu-3 further in the text.

We used the following Wnt pathway inhibitors:

1. clofazimine (15–17)
2. MU17, an analog of clofazimine obtained through medicinal chemistry-based optimization seeking improved solubility, potency, and selectivity of Wnt inhibition *in vitro* and *in vivo* (32)
3. F2-99, a proprietary pyrazole-based small molecule developed through our in-house HTS for upstream inhibitors of Wnt signaling in TNBC, possessing strong on-target *in vivo* effects in animal models without signs of animal toxicity (10)
4. ICG-001, a commercially available inhibitor of the Wnt signaling acting at the level of β-catenin-dependent transcription (33).

**FIG 1:**
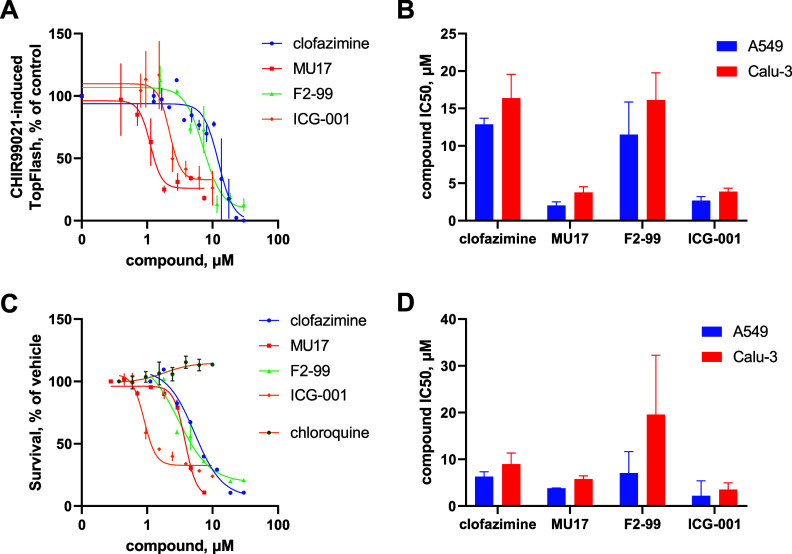
(A and B) Inhibition of Wnt signaling by the compounds used in the work. Representative curves obtained on A549 cells (A) and their quantification for both A549 and Calu-3 cell lines (B) are shown. (C and D) Inhibition of cell survival by the compounds used in the work. Representative curves obtained on A549 cells (C) and quantification for both A549 and Calu-3 cell lines in a 3-day MTT survival assay (D) are shown. Chloroquine was also tested in the survival assay to evaluate its suitability for further use as a positive control since it is a validated inhibitor of SARS-CoV-2 infection. The data are shown from *n* = 3 to 4 independent repeats.

We found that all tested compounds were effective as Wnt pathway inhibitors in the lung epithelial cell lines (Fig. 1A and B), and the IC_50_s of the Wnt pathway inhibition in lung cells corresponded to those previously observed in other cell lines (10, 16, 32, 33). We further tested the effects of these compounds on lung cell proliferation, confirming with all four drugs that Wnt signaling is required for the growth of these cancer cells (Fig. 1C and D), with the IC_50_s of growth inhibition and Wnt signaling suppression being in good correlation (Fig. 1B and D). Of the two cell lines used, Calu-3 cells were found to be generally more resistant to the compounds’ action than A549 cells.

**FIG 2:**
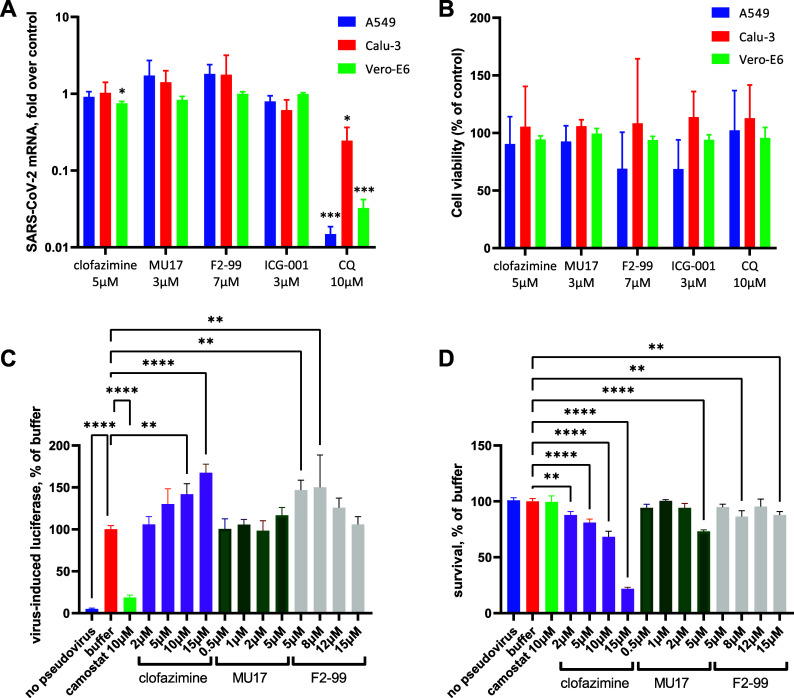
(A) qPCR evaluation of the viral titer over A549, Calu-3, and Vero-E6 cell lines in the presence of the indicated compounds, normalized to vehicle-treated cells upon infection at an MOI of 0.01. Note the log scale. (B) Parallel quantification of cell viability indicates no significant effect of the compounds on cell survival within the time of the assay. (C) A pseudoviral entry assay in A549 cells demonstrates that Wnt pathway inhibitors do not inhibit viral entry; clofazimine is instead stimulatory at higher concentrations. (D) At high clofazimine concentrations, the drug displayed certain cell toxicity. Statistical significance was assessed by one-way analysis of variance (ANOVA) for each cell line (A and B) or compound concentration set (C and D) relative to the vehicle for *n* = 3 independent repeats. The significance is shown as follows: *, *P* < 0.05; **, *P* < 0.01; ***, *P* < 0.001; ****, *P* < 0.0001.

We continued with the evaluation of the ability of Wnt inhibition to affect the viral cycle, with chloroquine—a known *in vitro* viral entry inhibitor (34)—used as a positive control. For the viral load quantification, we employed SYBR green-based quantitative PCR (qPCR), using a published set of primers specific for this variant (35), on day 3 postinfection. We used the compounds at concentrations around 2-fold lower than their IC_50_ in the survival assay (Fig. 1D) to avoid the distorting effects of cell death on the viral amplification cycle. The viability of the cells in the assay was controlled by 6-CFDA (6-carboxyfluorescein diacetate) live staining and subsequent quantification of the images by the built-in protocol in the software of the automated microscope. Additionally, in this set of experiments, we added Vero-E6 cells, the African green monkey kidney cell line used to model SARS-CoV-2 infection in previous studies (20, 21). Surprisingly, and quite opposite from the expectations from the previous reports (20, 21), we found that inhibition of Wnt signaling by the four distinct compounds, including clofazimine, did not result in any significant reduction of the viral RNA load in lung epithelium-derived lines: a ca. 25% reduction of the viral RNA titers could be seen upon application of clofazimine, but not other Wnt pathway inhibitors in Vero-E6 cells (Fig. 2A). Chloroquine, in contrast, was efficient in reducing SARS-CoV-2 infection in this system, more efficiently in A549 and Vero-E6 cells than in Calu-3 cells (note the logarithmic scale in Fig. 2A). None of the treatments, as intended, significantly affected the cells’ viability (Fig. 2B).

To reinforce these observations, we additionally performed a pseudoviral entry assay using a system similar to the one described (36), where lentivirus pseudotyped with the SARS-CoV-2 spike protein and encoding constitutively expressed firefly luciferase would serve as a model of the initial steps of the viral entry in A549 cells (see Materials and Methods). Using this assay, we found not only the absence of viral entry inhibition by the anti-Wnt compounds but instead even a certain increase in viral entry by clofazimine (Fig. 2C). It should be noted that a certain degree of toxicity is present at higher concentrations of clofazimine (Fig. 2D). However, since the viral load in Fig. 2C is normalized to cell survival, we must conclude that higher concentrations of clofazimine (but not other Wnt inhibitors) actually promote viral entry, in opposition to the prior observations (20), although the nature of this effect is unclear.

Thus, with two independent assays of SARS-CoV-2 infection in two lung epithelial cell lines and the Vero-E6 cells, we found that four different Wnt pathway inhibitors did not prevent the viral infection (with clofazimine displaying a marginal effect in Vero-E6 cells), in contrast with the expectations from the prior studies (20, 21). At the same time, the tested compounds efficiently inhibit the endogenously active Wnt pathway in lung cancer cells, accompanied by the suppression of lung cancer cell proliferation, in agreement with our studies on other cell types (10, 15–17).

Our findings call for caution when interpreting and generalizing the recent observations of the positive role of β-catenin in SARS-CoV-2 infection (21) and of the antiviral activity of clofazimine (20). The African green monkey kidney Vero-E6 cells have been used in both studies, and clofazimine and iCRT4, another Wnt pathway inhibitor (but not the JW67 Wnt pathway inhibitor), were found effective in suppressing SARS-CoV-2 infection in these cells. The same effect was also achieved by small interfering RNA (siRNA) against β-catenin in Vero-E6 cells. However, we find that in the lung cell lines, four distinct Wnt pathway inhibitors acting at different levels within the pathway fail to prevent the viral infection (with high concentrations of clofazimine actually promoting the viral entry), suggesting that the previously observed effects (20, 21) are likely cell type specific rather than general or relevant for lung epithelia. These discrepancies highlight the importance of the cell model chosen when studying SARS-CoV-2 infection. Interestingly, the famous anti-SARS-CoV-2 compound chloroquine is efficient at inhibiting viral entry in Vero-E6 cells and in A549 cells, but less so in Calu-3 cells (Fig. 2A), agreeing with earlier findings (34). At this stage, we also cannot exclude that the differences from the prior studies might also be attributed to the exact strain of the virus: the Delta variant in our study versus the HKU-001a strain in reference 20. (The exact variant used in the other study was not reported [21].) Other technical aspects, such as the use of antibiotics in the medium or the variability in the serum supplementation, might have also potentially contributed to different outcomes of the experiments in different laboratories.

Taking our data together, we conclude that the endogenous Wnt signaling is active in pulmonary epithelia and is required for cell survival in this tissue. However, our findings also suggest that, unlike as is the case for some other viral infections, the Wnt pathway is neither required nor involved in the SARS-CoV-2 infection in the lung, and pharmacological inhibition of this pathway with clofazimine or other compounds is not a promising way to develop treatments against the SARS-CoV-2 infection.

## MATERIALS AND METHODS

### Luciferase reporter assay.

The Wnt3a-stimulated Wnt pathway activity was assessed using the TopFlash reporter essentially as described previously (9, 32) in Calu-3 cells stably transfected and in A549-ACE2-TMPRSS2 cells transiently transfected with the reporter construct. The reporter cells were seeded at 500,000 cells/well in a 12-well plate in a final volume of 1 mL of Dulbecco’s modified Eagle’s medium –Ham’s F-12 medium (DMEM/F-12) supplemented with 10% fetal bovine serum (FBS). The cells were maintained at 37°C in 5% CO_2_ overnight for attachment. Subsequently, they were transfected with a plasmid encoding constitutively expressed *Renilla* luciferase under the cytomegalovirus (CMV) promoter alone or in a 1:1 mixture with the TopFlash plasmid (in the A549 cells) using 12 μg/mL of DNA and 40 μL/mL XtremeGENE HP reagent following the manufacturer’s protocol. The next day, the cells were detached and seeded at 10,000 cells/well in white opaque 384-well plates in 20 μL/well of fresh medium. After 24 h posttransfection, the medium was replaced by 10 μL of fresh medium containing the compound of interest and, after 1 h of preincubation, an additional 10 μL of thd medium supplemented with Wnt3a, purified as described previously (37, 38), at a final concentration of 2.5 μg/mL. Compounds were prepared by serial dilutions in DMSO and further diluted with the medium in amounts needed to obtain their final concentrations as indicated in the text and figures, maintaining the DMSO concentration of 0.5% at all assay points. After overnight incubation, the luciferase activity was measured as follows (39–41): the culture medium was completely removed from all wells of the plate, and the firefly and *Renilla* luciferase activities were detected sequentially in individual wells of a 384-well plate through injection of the corresponding measurement solutions and immediate reading (400-ms integration time) in an Infinite M Plex multifunctional plate reader equipped with an injection module. The data are presented as a two-point normalized signal with the Wnt3a-induced response set at 100% and the nonstimulated cells representing 0%.

### MTT assay.

For the MTT [3-(4,5-dimethyl-2-thiazolyl)-2,5-diphenyl-2H-tetrazolium bromide] assay, cells of the A549-ACE2-TMPRSS2, Calu-3, or Vero-E6 cell line were seeded at 1,000 cells/well in a transparent 384-well plate in a final volume of 20 μL/well. The cells were maintained in DMEM/F-12 containing 10% FBS at 37°C at 5% CO_2_ overnight. The next day, the medium was replaced by 40 μL fresh medium containing the indicated concentrations of compounds. Compound dilutions were prepared as described above. After incubation for 3 days, the medium in each well was replaced by 25 μL of 0.5 mg/mL thiazolyl blue solution in 1× phosphate-buffered saline (PBS), followed by incubation for 3 h at 37°C. Then, the solution was removed and 25 μL DMSO was added to each well. Absorbance at 510 nm was measured in a Tecan Infinite M Plex plate reader. Background readings were subtracted, and vehicle (DMSO alone) assay points were used as a control for the single-point normalization.

### SARS-Cov-2 infection and quantification.

For Calu-3, A549-ACE2-TMPRSS2, or Vero-E6 cells, 8,000 cells/well were seeded in a 96-well plate in 60 μL of DMEM/F-12 plus 10% FBS culture medium. The next day, the medium was replaced with 80 μL of DMEM/F-12 plus 2% FBS supplemented with the indicated amounts of the compounds. After 1 h of preincubation, an additional 20 μL of DMEM/F-12 plus 2% FBS with dilution of the Delta strain of SARS-CoV-2 was added into each well to create the final multiplicity of infection (MOI) of 0.01. The cells were incubated with virus for 3 days at 37°C at 5% CO_2_. To analyze viral titer, 20 μL medium was collected from each well and the viral RNA was purified using E.Z.N.A. viral RNA kit according to the manufacturer’s protocol.

The titer of the viral RNA was evaluated using a two-step protocol. Eluted RNA (70 μL) was dried in a SpeedVac and resuspended in 5 μL of water. Reverse transcription was performed by Moloney murine leukemia virus (M-MLV) reverse transcriptase using a forward primer (see below) according to the manufacturer’s protocol. This mixture was added as a template in qPCR at 0.5 μL/well, performed using PowerUp SYBR green master mix and Fast protocol in the StepOnePlus system with the following primers (China CDC [42]): forward primer CCCTGTGGGTTTTACACTTAA and reverse primer ACGATTGTGCATCAGCTGA.

To quantify the data, the threshold cycle (*C_T_*) of each sample was detected at an identical threshold and the relative quantity was calculated for each sample to the average of the DMSO-treated control. For all conditions, the concentration of DMSO was 0.1%.

### Pseudovirus entry assay.

The pseudovirus assay was performed as a service by Neurix SA (Geneva, Switzerland) using proprietary pseudolentivirus vectors expressing the SARS-Cov-2 spike protein with the luciferase reporter gene. A549-ACE2-TMPRSS2 cells were treated for 1 h with the indicated compounds. Then, the lentiviral vector coding for the luciferase reporter and expressing the SARS-CoV-2 spike protein with the D614G mutation was added for 6 h. Finally, the culture medium was changed, and cells were incubated for 2 additional days prior to measurements. Viral entry was measured by the normalized lentivirus-mediated luciferase signal, with WST8 cell viability displayed as light gray. The TMPRSS2 inhibitor camostat mesylate was used as an internal control. For all conditions, the concentration of DMSO was 0.1%.
